# Potent antiviral agents fail to elicit genetically-stable resistance mutations in either enterovirus 71 or Coxsackievirus A16

**DOI:** 10.1016/j.antiviral.2015.10.006

**Published:** 2015-12

**Authors:** James T. Kelly, Luigi De Colibus, Lauren Elliott, Elizabeth E. Fry, David I. Stuart, David J. Rowlands, Nicola J. Stonehouse

**Affiliations:** a School of Molecular and Cellular Biology, University of Leeds, Leeds, UK; b Division of Structural Biology, University of Oxford, Oxford, UK; c Diamond Light Source, Didcot, UK

**Keywords:** Hand foot and mouth disease, Capsid binding, Drug resistance, Fitness, VP1 pocket, VP1, Viral Protein 1, IC_50_, Half-maximal inhibitory concentration, CC_50_, Half maximal cytotoxic concentration, TCID_50_, Half-maximal tissue culture infective dose, PV, poliovirus, CVB, Coxsackie virus B, WT, Wild Type, MTT, 3-(4,5-Dimethylthiazol-2-Yl)-2,5-Diphenyltetrazolium Bromide, EV71, Enterovirus 71, CVA16, Coxsackie virus A16

## Abstract

Enterovirus 71 (EV71) and Coxsackievirus A16 (CVA16) are the two major causative agents of hand, foot and mouth disease (HFMD), for which there are currently no licenced treatments. Here, the acquisition of resistance towards two novel capsid-binding compounds, NLD and ALD, was studied and compared to the analogous compound GPP3. During serial passage, EV71 rapidly became resistant to each compound and mutations at residues I113 and V123 in VP1 were identified. A mutation at residue 113 was also identified in CVA16 after passage with GPP3. The mutations were associated with reduced thermostability and were rapidly lost in the absence of inhibitors. *In silico* modelling suggested that the mutations prevented the compounds from binding the VP1 pocket in the capsid. Although both viruses developed resistance to these potent pocket-binding compounds, the acquired mutations were associated with large fitness costs and reverted to WT phenotype and sequence rapidly in the absence of inhibitors. The most effective inhibitor, NLD, had a very large selectivity index, showing interesting pharmacological properties as a novel anti-EV71 agent.

## Highlights

• Resistance to each compound developed rapidly, mutations at residues I113 and V123 were identified.
• Resistance mutations were associated with a large fitness cost.
• The mutations resulted in reduced affinity for the pocket factor.
• The most effective inhibitor, NLD, had a very large therapeutic window, and has potential as a novel anti-EV71 agent.

Hand, foot and mouth disease (HFMD) usually manifests as a mild self-limiting childhood infection, causing sores on the hands, feet, mouth and buttocks, but can be associated with more serious symptoms, including fatal neurological/cardiovascular disorders. HFMD is usually caused by Enterovirus A species picornaviruses, especially enterovirus 71 (EV71) and Coxsackievirus A16 (CVA16), with EV71 more commonly associated with severe disease (Mcminn, 2003). EV71-mediated HFMD is therefore the major picornavirus-related public health problem in a post-poliovirus era and there are currently no clinically-approved therapeutic or prophylactic treatments.

WIN compounds and related molecules prevent receptor attachment/uncoating of a number of enteroviruses (Pevear et al., 1999). These compounds bind to a cavity in the capsid (the pocket in one of the viral capsid proteins, VP1) displacing hydrophobic lipids termed pocket factors. These are expelled upon receptor binding or uncoating, allowing the capsid to undergo a conformational change resulting in release of the RNA genome (Dang et al., 2014, Ren et al., 2013, Wang et al., 2012). The relatively high affinity between the pocket and WIN compounds prevents the conformational changes necessary for uncoating, increasing capsid stability (Rotbart et al., 1998) and can be effective at preventing infection in culture (Pevear et al., 1999, Shia et al., 2002, Benschop et al., 2015), and murine models (Groarke and Pevear, 1999, Liu et al., 2012). One compound, Vapendavir (BTA798), has shown efficacy in asthmatic patients with human rhinovirus (HRV) infections in phase II trials (Feil et al., 2012).

A related compound, Pleconaril, was used as a model to design a new class of pyridyl imidazolidinones, (IC_50_ values against EV71 of 0.001–25 μM), the most potent of which was termed GPP3 (Ke and Lin, 2006, Shia et al., 2002). Crystallographic analysis of EV71 (Plevka et al., 2012, Wang et al., 2012) in complex with four different pyridyl imidazolidinones, combined with computational methods including quantum mechanics–enhanced ligand docking were used to develop two new compounds (NLD and ALD), based upon GPP3 (De Colibus et al., 2014). NLD was shown to have more than an order of magnitude greater potency against EV71 than GPP3, IC_50_ = 25 pM. GPP3 and the new derivative compounds also had anti-CVA16 activity.

**Fig. 1:**
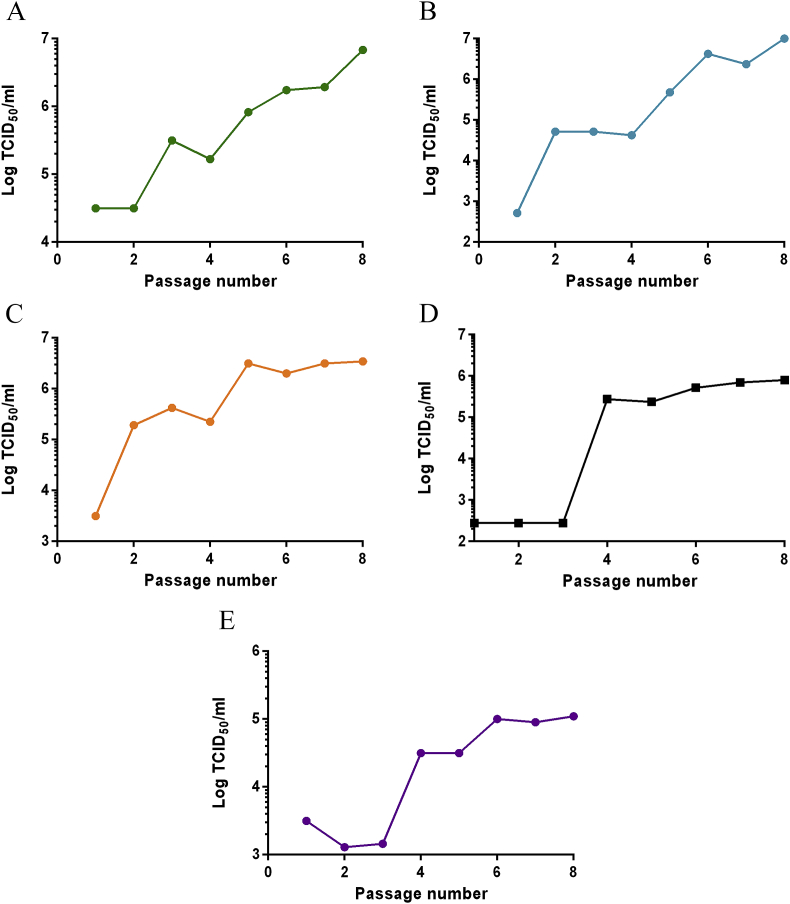
Generation of resistant isolates. WT EV71 was passaged in the presence of either (A) 0.1 nM NLD, (B) 0.9 nM GPP3, (C) 80 nM ALD or, (D) a combination of 0.1 nM NLD and 0.9 nM GPP3, (E) WT CVA16 was passaged in the presence of 20 nM GPP3. Each isolate was passaged a total of 8 times and after each passage a sample was titrated in the presence of the selecting concentration of compound. CVA16 isolates resistant to NLD or ALD were not selected.

To identify mutations associated with resistance, EV71/CVA16 were passaged eight times in the presence of these compounds at concentrations that reduced the TCID_50_ values by over 99.9% (see supplementary information). Virus titres in the presence of the compounds rapidly rose to WT-equivalent levels (∼1 × 10^7^ TCID_50_/ml for EV71 and ∼1 × 10^5^ TCID_50_/ml for CVA16), indicating the acquisition of resistance (Fig. 1). Sequencing revealed three different VP1 mutants in EV71 (I113L, I113M, I113M/V123I) and one in CVA16 (L113F) (Table 1). Virus passaged in a combination of NLD/GPP3 over 30 passages maintained the I113M/V123I mutations (n = 1).

**Fig. 2:**
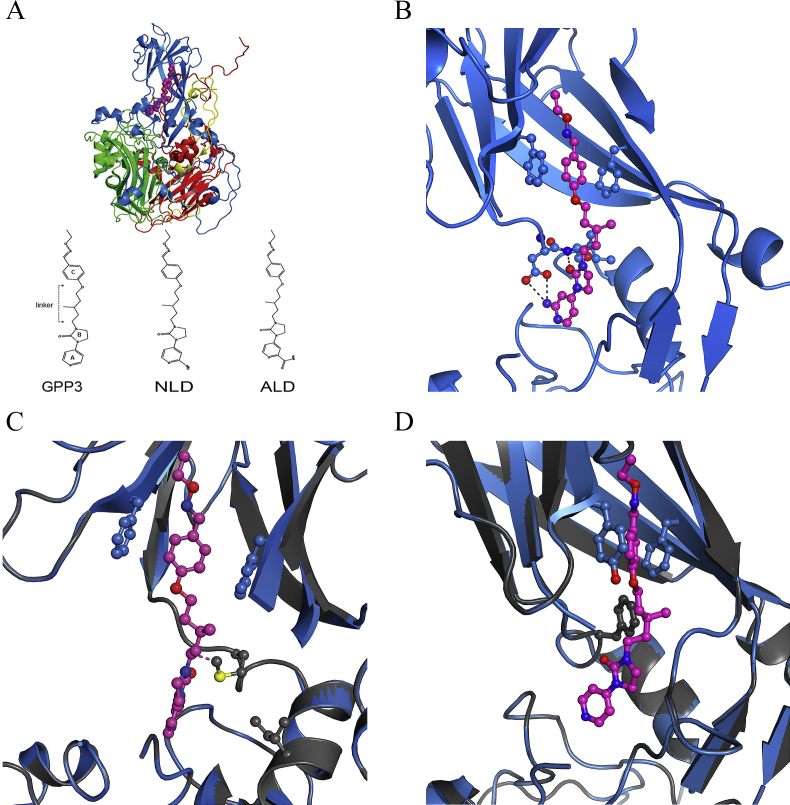
(A) Structure of EV71 protomer in complex with NLD. Icosahedral protomeric unit of EV71 (PDBid 4CEY), viewed from the inside of the capsid. VP1, blue, VP2, green, VP3 red, VP4 yellow, NLD magenta. Protein shown in cartoon representation, NLD as spheres, with the chemical structures of GPP3, NLD and ALD molecules reported below. (B) Structure of EV71 pocket in complex with NLD. EV71 (PDBid 4CEY) showing a close-up of the VP1 (blue) pocket with NLD (magenta) in-place. The side-chains of F135, F155, D112 and I113 and NLD are shown as ball-and-sticks. Hydrogen bonds are shown as dashed (C) Predicted structure of inhibitor resistant mutant in complex with NLD. EV71 (PDBid 4CEY) in blue, with VP1 model generated by Rossetta shown in grey. The two mutated side chains I113M and V123I are shown as grey balls-and-sticks. The distance between the M113 side chain and NLD linker, corresponding to 2.1 Å, is shown as a dotted line. (D) Structure of CVA16 (PDBid:5ABJ) in complex with GPP3. Close-up view of the VP1 pocket (in blue) with GPP3 bound (magenta). The side chains of Y135 and F155 are shown as blue ball-and-sticks. The predicted structure of the inhibitor-resistant mutant in complex with GPP3 is shown in grey with the L113F mutation in grey ball-and-sticks. In all ball-and-stick representation, nitrogen atoms are blue, oxygen red and sulphur yellow. Sequencing shows a change to M or F could be achieved by a single point mutation for both EV71 and CVA16. Comparison with the NCBI gene bank database revealed that none of the 5830 EV71 or 248 CVA16 sequences deposited contained any combination of these mutations.

The crystal structure of EV71 in complex with NLD enabled visualisation of the NLD binding site within the protomeric unit of the capsid, this showed that all interactions of NLD are with VP1 (Fig. 2A) (De Colibus et al., 2014). Fig. 2B and C shows the interactions close-up, with key stabilising interactions highlighted and the location of the resistance mutations in EV71, respectively. Mutations I113M and V123I are located on the inside of the VP1 pocket and I113 is one of the residues involved in compound binding (Fig. 2B). Fig. 2D shows the location of the resistance mutation for CVA16 in the context of the adjacent GPP3 molecule.

Studies with other enteroviruses have documented many mutations associated with resistance to a variety of pocket-binding compounds (see Supplementary Information). Generally, resistant viruses have acquired mutations that interfere with the correct placement of the inhibitors in the binding pocket (Liu et al., 2012). To test this in the EV71/CVA16 resistant-isolates, *in silico* folding energy predictions resulting from the mutations were performed using Rosetta (Fowler et al., 2010, Kellogg et al., 2011, Tyka et al., 2011) on VP1 subunits. The difference in the lowest free energy of folding (ΔΔG_folding_) between the WT and EV71 I113M or V123I mutants was +0.73 and + 0.98 kcal/mol, respectively. The combination of both mutations gave ΔΔG_folding_ of ∼+1.1 kcal/mol per VP1 (note that this value should be multiplied by 60 to reflect the number of VP1 molecules per capsid), suggesting that the mutant virus capsid is less stable than WT. The mutations I113M and V123I appeared to cause a shrinking of the VP1 pocket, with the methionine residue pointing inside, suggesting a steric clash with the pocket factor. Thus, the distance between the carbon atom of the methyl group of the methionine side chain and a carbon atom in the linker of NLD is 2.1 Å (Fig. 2C), smaller than the sum of the Van der Waals radii and leading to a repulsive interaction. However, the ΔΔG_folding_ for the CVA16 mutant was −1.7 kcal/mol, suggesting that the mutant is more stable than WT (not taking pocket factor into account). The Rosetta structures are shown in grey in Fig. 2C and D.

**Fig. 3:**
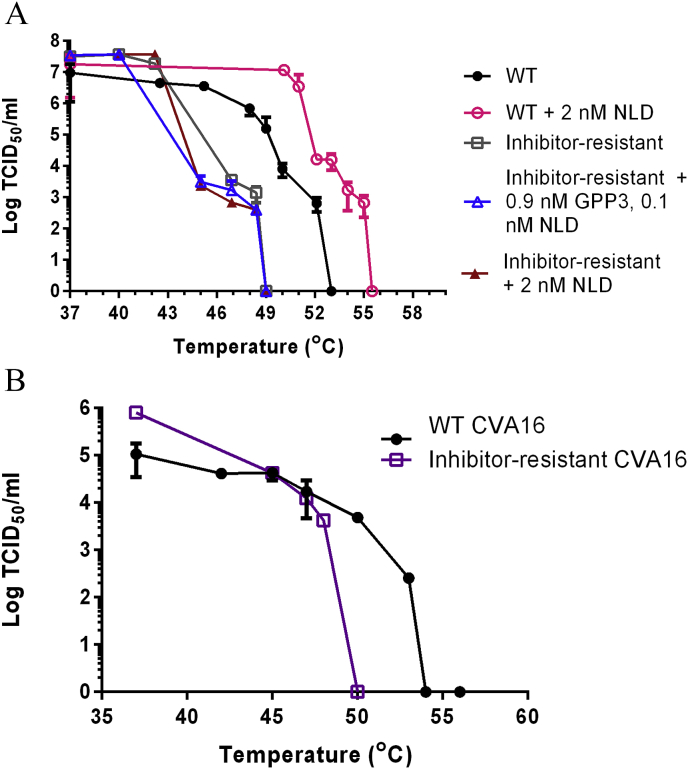
Inhibitor-resistant mutants are more thermolabile than WT virus. (A) Thermolability curves of EV71 WT (filled circle) and inhibitor-resistant EV71 (open square), WT EV71 in the presence of 2 nM NLD (open circle), inhibitor-resistant EV71 in the presence of 0.9 nM GPP3 and 0.1 nM NLD (open triangle) and 2 nM NLD (filled triangle) (B) Thermolability curves of WT CVA16 (filled circle) and inhibitor-resistant CVA16 (open square). All samples were heated at a range of temperatures for 30 min using a thermocycler, prior to titration by TCID_50_ assay. Samples containing NLD were first diluted to a level at which the inhibitor has no effect before titration. Titrations were carried out in triplicate and error was measured using standard deviation.

It has been reported that pocket-binding compounds increased thermostability of WT PV and HRVs, while inhibitor-resistant isolates with mutations in the pocket were often more thermolabile than their untreated WT counterpart (Katpally et al., 2007, Mosser et al., 1994, Shepard et al., 1993). The thermostabilities of WT EV71 and inhibitor-resistant mutant virus selected in the presence of NLD/GPP3 were therefore evaluated. WT EV71 (heated with NLD) was tolerant of temperatures ∼6 °C higher before detectable inactivation and 2.5 °C higher before complete inactivation occurred, compared to virus in the absence of NLD (Fig. 3A). In contrast, the thermostability of inhibitor-resistant virus was not increased in the presence of NLD or by a combination of GPP3 and NLD. In addition, the inhibitor-resistant mutant virus was more thermolabile than WT, with initial inactivation occurring at a temperature 3–4 °C lower. Similar results were obtained with inhibitor-resistant CVA16 (Fig. 3B).

These data suggest that the I113M/V123I mutation prevented compound binding as the thermostability of the resistant isolates was not increased in the presence of NLD. The bulkier side chains of the I113M and L113F mutations are predicted to point directly into the pocket, reducing the space available for inhibitor binding and the V123I mutation reduces this further. The ΔΔG_folding_ calculations predicted that there is a difference in the stability of viruses carrying the resistance mutations (in the absence of pocket factor), however, it is likely that the mutations also have a deleterious effect on the binding of the natural pocket factor, which is known to be a virion stabiliser. Similar results have been reported for WIN51711-resistant PV3 and WIN52035-2-resistant HRV14, with mutations identified in the VP1 pocket (Mosser et al., 1994, Shepard et al., 1993).

**Fig. 4:**
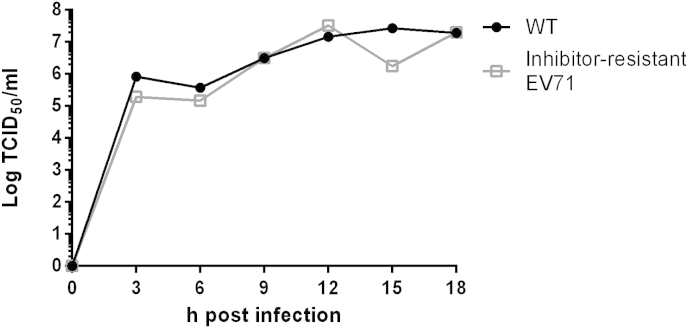
One step growth curves comparing inhibitor-resistant and WT EV71. WT (filled circle) and inhibitor-resistant EV71 selected in the presence of 0.1 nM NLD and 0.9 nM GPP3 (open square) were used to infect six wells each of a 96 well plate at an MOI of 10. Every 3 h the supernatant and cells of a well from each isolate were removed, and freeze-thawed to lyse the cells. These samples were then titrated by TCID_50_ assay.

To assess whether the mutations affected growth kinetics, one-step growth curves were performed with WT EV71 and a resistant isolate. These showed no significant difference between the WT and the resistant virus (Fig. 4). The phenotype of the mutant viruses described here is consistent with previous observations with other picornaviruses (Groarke and Pevear, 1999, Heinz et al., 1989, Lacroix et al., 2014, Liu et al., 2012, Mosser et al., 1994, Salvati et al., 2004). However, it should be noted that a small difference in kinetics of virus resistant to BPR0Z-19 has been reported (Shih et al., 2004).

**Fig. 5:**
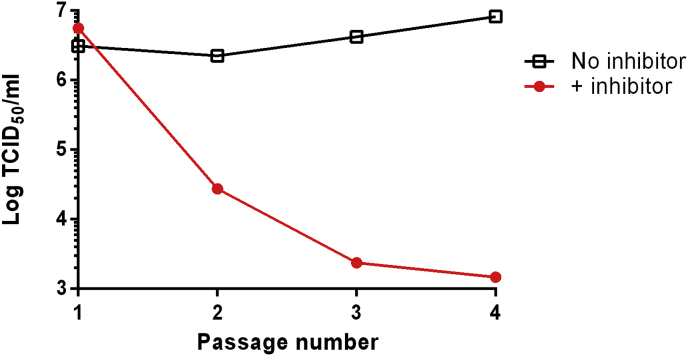
Inhibitor-resistant isolates are genetically unstable. A NLD/GPP3 inhibitor-resistant isolate selected with a combination of 0.1 nM NLD and 0.9 nM GPP3 was passaged four times in the absence of inhibitor. Virus from each passage was titrated in the presence (filled circles) or absence of inhibitors (open squares) by TCID_50_ assay.

Mutations are often associated with a fitness cost, therefore the genetic stability of inhibitor-resistant EV71 was tested by passage in the absence of inhibitors. The titre in the presence of inhibitor dropped dramatically after just one passage. After two further passages the titre became equivalent to the WT in the presence of the inhibitors (Fig. 5). Sequencing confirmed that the virus had reverted to a WT genotype (n = 1), indicating a strong selection pressure for reversion. However, it should be noted that as this experiment was performed with a pool of viruses, it is not formally possible to distinguish between the amplification of residual WT virus in the population and back-mutation of selected mutant virus.

**Fig. 6:**
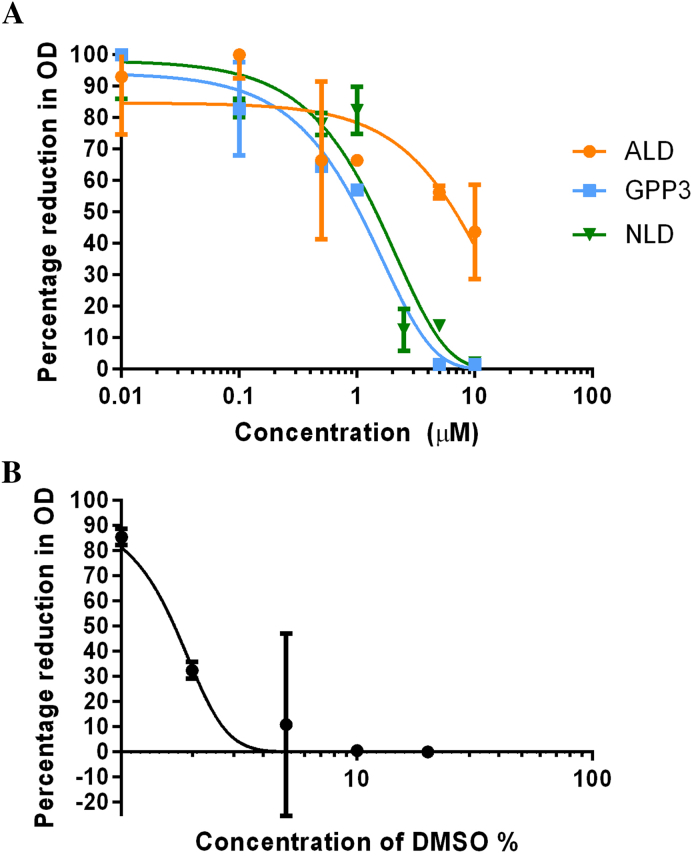
Toxicity of NLD, GPP3 and ALD to Vero cells. Vero cells were incubated with a range of concentrations of NLD (closed inverted triangle), GPP3 (filled square), ALD (filled circle) (A) or DMSO (B) for seven days. MTT assays were then performed and the CC_50_ was derived from the OD (692 nm) by non-linear regression. Each point represents an average of three independent experiments and standard deviation values are shown. The CC_50_ values for the three inhibitors were 1.6 μM, 1.0 μM and 7.0 μM respectively. DMSO (1%) had no apparent cytotoxic effect.

Fitness costs appear to be common in pocket inhibitor-resistant enteroviruses, with Pleconaril-resistant CVB3 and V-073-resistant PV causing reduced virulence in murine models (Groarke and Pevear, 1999, Kouiavskaia et al., 2011). Similarly, Pleconaril-resistant HRV B isolated from patients was shown to be non-pathogenic and was associated with a greatly reduced viral load (Pevear et al., 2005). The avirulent PV Sabin strains are also thermolabile and other thermolabile EV71 mutants have been shown to have reduced pathogenesis in Cynomolgus monkeys (Arita et al., 2005). Given the apparent fitness cost of these mutations, resistance may not pose an obstacle to the therapeutic use of pocket-binding compounds. Indeed, assessment of the toxicity of the three compounds showed NLD to have a large selectivity index (Fig. 6, Table 2). However, further work will be necessary in order to evaluate if inhibitor-resistant EV71/CVA16 mutants are less virulent.

In conclusion, we have described the selection and characterisation of resistance mutations of EV71 in the presence of three pocket-binding inhibitory compounds. We modelled these mutations in the VP1 pocket to explain their effect on the binding mode of the pocket factor and calculated the ΔΔG_folding_ of these mutants. The *in silico* predictions were in agreement with some of the observed properties of the viruses *in vitro*. Furthermore we found that the most effective EV71 pocket-binding inhibitor, NLD, has the greatest selectivity index, showing interesting pharmacological properties as a novel anti-EV71 agent.

## Funding

This work was supported by a University of Leeds PhD studentship from Sanofi Pasteur and work at Oxford was funded by the Medical Research Council (G100099).

## Transparency declarations

Nothing to declare.

## Figures and Tables

**Table 1:** Resistance mutations. Mutations identified in EV71/CVA16 after passage in the presence of NLD, GPP3, ALD or a combination of NLD/GPP3 (see Fig. 1). CVA16 isolates resistant to NLD or ALD were not selected.

Virus/Compound	Mutation(s)	Number sequenced
EV71/NLD	I113M (62%), I113L (31%), I113M/V123I (7%)	n = 13
EV71/GPP3	I113M/V123I	n = 4
EV71/ALD	I113M/V123I	n = 1
EV71/NLD + GPP3	I113M/V123I	n = 12
CVA16/GPP3	L113F	n = 10

**Table 2:** Selectivity index. The selectivity index of each compound was calculated by dividing the CC_50_ by the IC_50_ value. IC_50_ values are for strain EV71 Genotype B2, Strain MS742387, taken from De Colibus et al. (2014), which was grown in Vero cells.

	NLD	GPP3	ALD
CC_50_ (Cell toxicity)	1600 nM	1000 nM	7000 nM
IC_50_ (Virus inhibition)	0.025 nM	0.39 nM	8.7 nM
Selectivity Index (CC_50_: IC_50_)	6 × 10^4^	2.6 × 10^3^	8 × 10^2^

## References

[bib1] Arita M., Shimizu H., Nagata N., Ami Y., Suzaki Y., Sata T., Iwasaki T., Miyamura T. (2005). Temperature-sensitive mutants of enterovirus 71 show attenuation in cynomolgus monkeys. J. Gen. Virol..

[bib2] Benschop K.S., van der Avoort H.G., Duizer E., Koopmans M.P. (2015). Antivirals against enteroviruses: a critical review from a public-health perspective. Antivir. Ther..

[bib3] Dang M., Wang X., Wang Q., Wang Y., Lin J., Sun Y., Li X., Zhang L., Lou Z., Wang J., Rao Z. (2014). Molecular mechanism of SCARB2-mediated attachment and uncoating of EV71. Protein Cell..

[bib4] De Colibus L., Wang X., Spyrou J.A.B., Kelly J., Ren J., Grimes J., Puerstinger G., Stonehouse N., Walter T.S., Hu Z., Wang J., Li X., Peng W., Rowlands D.J., Fry E.E., Rao Z., Stuart D.I. (2014). More-powerful virus inhibitors from structure-based analysis of HEV71 capsid-binding molecules. Nat. Struct. Mol. Biol..

[bib5] De Colibus L., Wang X., Tijsma A., Neyts J., Spyrou J.A.B., Ren J., Grimes J.M., Puerstinger G., Leyssen P., Fry E.E., Rao Z., Stuart D.I. (2015). Structure elucidation of Coxsackievirus A16 in complex with GPP3 informs a systematic review of highly potent capsid binders to enteroviruses. PLoS Path.

[bib6] Feil S.C., Hamilton S., Krippner G.Y., Lin B., Luttick A., Mcconnell D.B., Nearn R., Parker M.W., Ryan J., Stanislawski P.C., Tucker S.P., Watson K.G., Morton C.J. (2012). An orally available 3-Ethoxybenzisoxazole capsid binder with clinical activity against human rhinovirus. ACS Med. Chem. Lett..

[bib7] Fowler D.M., Araya C.L., Fleishman S.J., Kellogg E.H., Stephany J.J., Baker D., Fields S. (2010). High-resolution mapping of protein sequence- function relationships. Nat. Methods.

[bib8] Groarke J.M., Pevear D.C. (1999). Attenuated virulence of pleconaril-resistant coxsackievirus B3 variants. JID.

[bib9] Heinz B.A., Rueckert R.R., Shepard D.A., Dutko F.J., Mckinlay M.A., Fancher M., Rossmann M.G., Badger J., Smith T.J. (1989). Genetic and molecular analyses of that are resistant to an antiviral compound. Genetic and molecular analyses of spontaneous mutants of human rhinovirus 14 that are resistant to an antiviral compound. J. Virol..

[bib10] Katpally U., Smith T.J., Rhinovirus H. (2007). Pocket factors are unlikely to play a major role in the life cycle of human rhinovirus pocket factors are unlikely to play a major role in the life cycle of. J. Virol..

[bib11] Ke Y., Lin T. (2006). Modeling the ligand - receptor interaction for a series of inhibitors of the capsid protein of enterovirus 71 using several three-dimensional quantitative structure – activity relationship techniques. J. Med. Chem..

[bib12] Kellogg E.H., Leaver-fay A., Baker D. (2011). Role of conformational sampling in computing mutation-induced changes in protein structure and stability. Proteins.

[bib13] Kouiavskaia D.V., Dragunsky E.M., Liu H., Oberste M.S., Collett M.S., Chumakov K.M. (2011). Original article immunological and pathogenic properties of poliovirus variants selected for resistance to antiviral drug V-073. Antivir. Ther..

[bib14] Lacroix C., Qluerol-audı J., Roche M., Franco D., Froeyen M., Guerra P., Terme T., Vanelle P., Verdaguer N., Neyts J., Leyssen P. (2014). A novel benzonitrile analogue inhibits rhinovirus replication. J. Antimicrob. Chemother..

[bib15] Liu H., Roberts J.A., Moore D., Anderson B., Pallansch M.A., Pevear D.C., Collett M.S., Oberste M.S. (2012). Characterization of poliovirus variants selected for resistance to the antiviral compound V-073. Antimicrob. Agents Chemother..

[bib16] Mcminn P.C. (2003). Enterovirus 71 in the Asia-Pacific region: an emerging cause of acute neurological disease in young children. Neurol. J. Southeast Asia.

[bib17] Mosser A.G., Sgro J., Rueckert R.R. (1994). Distribution of drug resistance mutations in type 3 poliovirus identifies three regions involved in uncoating functions. Distribution of drug resistance mutations in type 3 poliovirus identifies three regions involved in uncoating Functions. J. Virol..

[bib18] Pevear D.C., Hayden F.G., Demenczuk T.M., Barone L.R., Mckinlay M.A., Collett M.S. (2005). Relationship of pleconaril susceptibility and clinical outcomes in treatment of common colds caused by rhinoviruses relationship of pleconaril susceptibility and clinical outcomes in treatment of common colds caused by rhinoviruses. Antimicrob. Agents Chemother..

[bib19] Pevear D.C., Tull T.M., Seipel M.E., Groarke M., Groarke J.M. (1999). Activity of pleconaril against enteroviruses. Act. Pleconaril Against Enteroviruses.

[bib20] Plevka P., Perera R., Cardosa J., Kuhn R.J., Rossmann M.G. (2012). Crystal structure of human enterovirus 71. Science.

[bib21] Ren J., Wang X., Hu Z., Gao Q., Sun Y., Li X., Porta C., Walter T.S., Gilbert R.J., Zhao Y., Axford D., Williams M., Mcauley K., Rowlands D.J., Yin W., Wang J., Stuart D.I., Rao Z., Fry E.E. (2013). Picornavirus uncoating intermediate captured in atomic detail. Nat. Commun..

[bib22] Rotbart H.A., Connell J.F.O., Mckinlay M.A. (1998). Treatment of human enterovirus infections. Antivir. Res..

[bib23] Salvati A.L., Dominicis A., De Tait S., Canitano A., Lahm A., Fiore L. (2004). Mechanism of action at the molecular level of the antiviral drug 3 ( 2H ) -Isoflavene against type 2 poliovirus mechanism of action at the molecular level of the antiviral drug 3 ( 2H ) -Isoflavene against type 2 poliovirus. Antimicrob. Agents Chemother..

[bib24] Shepard D. a, Heinz B. a, Rueckert R.R. (1993). WIN 52035-2 inhibits both attachment and eclipse of human rhinovirus 14. J. Virol..

[bib25] Shia K., Li W., Chang C., Hsu M., Chern J., Leong M.K., Tseng S., Lee C., Lee Y., Chen S., Peng K., Tseng H., Chang Y., Tai C., Shih S. (2002). Design, synthesis, and structure - activity relationship of pyridyl imidazolidinones: a novel class of potent and selective human enterovirus 71. J. Med. Chem..

[bib26] Shih S., Tsai M., Tseng S., Won K., Shia K., Li W., Chen G., Lee C., Lee Y., Peng K., Chao Y. (2004). Mutation in enterovirus 71 capsid protein VP1 confers resistance to the inhibitory effects of pyridyl imidazolidinone mutation in enterovirus 71 capsid protein VP1 confers resistance to the inhibitory effects of pyridyl imidazolidinone. Antimicrob. Agents Chemother..

[bib27] Tyka M.D., Keedy D. a, André I., Dimaio F., Song Y., Richardson D.C., Richardsonb J.S., Baker D. (2011). Landscape mapping. J. Mol. Biol..

[bib28] Wang X., Peng W., Ren J., Hu Z., Xu J., Lou Z., Li X., Yin W., Shen X., Porta C., Walter T.S., Evans G., Axford D., Owen R., Rowlands D.J., Wang J., Stuart D.I., Fry E.E., Rao Z. (2012). A sensor-adaptor mechanism for enterovirus uncoating from structures of EV71. Nat. Struct. Mol. Biol..

