# Supplementary material for: Potent antiviral agents fail to elicit genetically-stable resistance mutations in either enterovirus 71 or Coxsackievirus A16
Source: Antiviral Res. 2015 Dec;124:77–82. doi: 10.1016/j.antiviral.2015.10.006 (PMC4678291; doi:10.1016/j.antiviral.2015.10.006)
Supplement: Supplementary file 1 [file mmc1.docx]

**Supplementary information**

# Material and Methods

## Virus and cells

Vero cells were grown in DMEM (Dulbecco's Modified Eagle Medium) supplemented with 10% FBS (Fetal Bovine Serum), penicillin and streptomycin at 37^o^C and 5% CO_2_. EV71 Genotype B2, Strain MS742387 was obtained from SingVax. CVA16 strain G-10 was supplied by Peter Simmonds. Virus was propagated in the same medium but without FBS.

## Virus Titration

Viruses were titrated on Vero cells by TCID_50_ assay (Reed and Muench, 1938). Viral samples were serially diluted (10^−1^ to 10^−8^) and added to Vero cells grown in 96-well plates. Each dilution was replicated 10x along with two control wells (no virus). Plates were incubated for seven (EV71) or nine (CVA16) days at 37°C and then stained with crystal violet and the cytopathic effect (CPE) was evaluated. Each experiment was repeated 3x.

## Selection of resistance

WT virus was grown in the presence of each compound at a concentration able to reduce the TCID_50_ value by over 99.9% i.e. 0.1 nM NLD, 0.9 nM GPP3 and 80 nM ALD for EV71 and 20 nM GPP3 for CVA16. Once 100% cell death was achieved, viruses were titrated and passaged in the presence of the same concentration of compound, until 100% cell death was achieved. This was repeated for eight passages.

RNA was extracted from the crude viral lysates and subjected to RT-PCR amplification (primer sequences available on request) prior to sequencing.

## Folding calculations

EV71 in complex with NLD (PDBID:4CEY) and CVA16 in complex with GPP3 (De Colibus., et al 2015) were used. Differences in fold­ing free energy (ΔΔ*G*_folding_) were performed by Rosetta, relaxing the VP1 subunit structure with coordinate constraints, and applying monomer ΔΔ*G*_folding_ calculation without sampling alternative backbone conformations (Fowler et al., 2010; Kellogg et al., 2011; Tyka et al., 2011). The ΔΔ*G*_folding_ calculation was performed 50 times for each simulation.. The pre­dicted folding free energy is the average of the lowest scor­ings, each of them obtained by five individual simulations. Structural superposition was performed in Coot (Emsley et al., 2010) using SSM superpose (Krissinel and Henrick., 2004). Figures were prepared with PyMOL (<http://www.pymol.org/>).

## One-step growth curves and thermolability assays

One-step growth curves of WT and inhibitor-resistant EV71 selected in the presence of NLD/GPP3 were carried out in Vero cell monolayers in 96-well plates. Monolayers were infected at a multiplicity of infection (MOI) of 10. After 3h media was removed and cells washed with PBS to remove unbound virus. Then every 3h until 18h post-infection, cells were removed by scraping from one well (for each isolate). Cells were freeze-thawed 3x and titrated in the absence of compound.

For thermolability assays, crude lysate was heated for 30 mins at various temperatures using a thermocycler, prior to titration as above.

## MTT Assay

3-(4,5-[di](http://en.wikipedia.org/wiki/Di-)[methyl](http://en.wikipedia.org/wiki/Methyl)[thiazol](http://en.wikipedia.org/wiki/Thiazole)-2-yl)-2,5-di[phenyl](http://en.wikipedia.org/wiki/Phenyl)tetrazolium bromide (MTT) assays were carried out by incubating Vero cells in the presence of each compound (at a range of concentrations) for seven days. A Promega CellTiter 96 kit was used following the manufacturer’s instructions.

**Supplementary Table 1. Pocket-binding inhibitor resistance mutations seen in Enterovirus species.**

**References**

Benschop, K.S., van der Avoort H.G., Duizer E., Koopmans, M.P., 2015. Antivirals against enteroviruses: a critical review from a public-health perspective. Antivir Ther. 20, 121-130

De Colibus, L., Wang, X., Tijsma, A., Neyts, J., Spyrou, J.A.B., Ren, J., Grimes, J.M., Puerstinger, G., Leyssen, P., Fry, E.E., Rao, Z., Stuart, D.I., 2015 Structure elucidation of Coxsackievirus A16 in complex with GPP3 informs a systematic review of highly potent capsid binders to enteroviruses. PLoS Path. In press

Emsley, P., Lohkamp, B., Scott, W. G. & Cowtan, K. 2010. Features and development of Coot. Acta Crystallogr D 66, 486-501.

Fowler, D.M., Araya, C.L., Fleishman, S.J., Kellogg, E.H., Stephany, J.J., Baker, D., Fields, S., 2010. High-resolution mapping of protein sequence- function relationships. Nat. Methods 7, 741–746.

Groarke, J.M., Pevear, D.C., 1999. Attenuated Virulence of Pleconaril-Resistant Coxsackievirus B3 Variants. JID 179, 1538–1541.

Heinz, B.A., Rueckert, R.R., Shepard, D.A., Dutko, F.J., Mckinlay, M.A., Fancher, M., Rossmann, M.G., Badger, J., Smith, T.J., 1989. Genetic and molecular analyses of that are resistant to an antiviral compound . Genetic and Molecular Analyses of Spontaneous Mutants of Human Rhinovirus 14 That Are Resistant to an Antiviral Compound. J. Virol. 63, 2476-2485.

Kellogg, E.H., Leaver-fay, A., Baker, D., 2011. Role of conformational sampling in computing mutation-induced changes in protein structure and stability. Proteins 79, 830–838.

Krissinel, E., Henrick, K., 2004. Secondary-structure matching (SSM), a new tool for fast protein structure alignment in three dimensions. Acta Crystallogr D Biol Crystallogr 60, 2256-2268.

Lacroix, C., Qluerol-audı, J., Roche, M., Franco, D., Froeyen, M., Guerra, P., Terme, T., Vanelle, P., Verdaguer, N., Neyts, J., Leyssen, P., 2014. A novel benzonitrile analogue inhibits rhinovirus replication. J. Antimicrob. Chemother. 69, 2723–2732.

Ledford R.M., Collett M.S., Pevear D.C., 2005. Insights into the genetic basis for natural phenotypic resistance. Antiviral Res. 68, 135-138

Liu, H., Roberts, J.A., Moore, D., Anderson, B., Pallansch, M.A., Pevear, D.C., Collett, M.S., Oberste, M.S., 2012. Characterization of Poliovirus Variants Selected for Resistance to the Antiviral Compound V-073. Antimicrob. Agents Chemother. 56, 5568–5574.

Mosser, A.G., Sgro, J., Rueckert, R.R., 1994. Distribution of drug resistance mutations in type 3 poliovirus identifies three regions involved in uncoating functions . Distribution of Drug Resistance Mutations in Type 3 Poliovirus Identifies Three Regions Involved in Uncoating Functions. J. Virol. 68, 8193-8201.

Reed, L., Muench, H., 1938. A simple method of estimating fifty percent endpoints. Am. J. Hyg. 27, 493–497.

Salvati, A.L., Dominicis, A. De, Tait, S., Canitano, A., Lahm, A., Fiore, L., 2004. Mechanism of Action at the Molecular Level of the Antiviral Drug 3 ( 2H ) -Isoflavene against Type 2 Poliovirus Mechanism of Action at the Molecular Level of the Antiviral Drug 3 ( 2H ) -Isoflavene against Type 2 Poliovirus. Antimicrob. Agents Chemother. 48, 2233-2243.

Shia, K., Li, W., Chang, C., Hsu, M., Chern, J., Leong, M.K., Tseng, S., Lee, C., Lee, Y., Chen, S., Peng, K., Tseng, H., Chang, Y., Tai, C., Shih, S., 2002. Design , Synthesis , and Structure - Activity Relationship of Pyridyl Imidazolidinones : A Novel Class of Potent and Selective Human Enterovirus 71. J. Med. Chem 45, 1644–1655.

Shepard, D. a, Heinz, B. a, Rueckert, R.R., 1993. WIN 52035-2 inhibits both attachment and eclipse of human rhinovirus 14. J. Virol. 67, 2245–2254.

Tyka, M.D., Keedy, D. a, André, I., Dimaio, F., Song, Y., Richardson, D.C., Richardsonb, J.S., Baker, D., 2011. Landscape Mapping. J Mol Biol 405, 607–618.
