# Supplementary material for: Potent antiviral agents fail to elicit genetically-stable resistance mutations in either enterovirus 71 or Coxsackievirus A16
Source: Antiviral Res. 2015 Dec;124:77–82. doi: 10.1016/j.antiviral.2015.10.006 (PMC4678291; doi:10.1016/j.antiviral.2015.10.006)
Supplement: Supplementary file 2 [file mmc2.docx]

| Virus | Mutation | Inhibitor | Ref |
| --- | --- | --- | --- |
| EV71 | I1113M | NLD |  |
|  | I1113L | NLD |  |
|  | I1113M/L1123V | NLD,GPP3,ALD |  |
|  | V1192M | BPR0Z-194 | Shih et al. 2004 |
|  |  |  |  |
| CVA16 | L1113F | GPP3 |  |
|  |  |  |  |
| Polio 1 | A1088T/A3024V | V-073 | Liu et al 2012 |
|  | P1161S | V-073 | Liu et al 2012 |
|  | I1183T | V-073 | Liu et al 2012 |
|  | I1194M | V-073 | Liu et al 2012 |
|  | I1194F | V-073 | Liu et al 2012 |
|  | A3024V | V-073 | Liu et al 2012 |
|  |  |  |  |
| Polio 2 | D1131V | 3(2H)-Isoflavene | Salvati et al 2004 |
|  | I1194M | 3(2H)-Isoflavene/V073 | Salvati et al 2004 /Liu et al 2012 |
|  | I1194F | V073 | Liu et al 2012 |
|  | N1053S | 3(2H)-Isoflavene | Salvati et al 2004 |
|  | K4058G | 3(2H)-Isoflavene | Salvati et al 2004 |
|  |  |  |  |
| Polio 3b | M1105T | WIN51711 | Mosser et al. 1994 |
|  | F1237L | V-073 | Mosser et al. 1994/Liu et al 2012 |
|  | D1129V | WIN51711 | Mosser et al. 1994 |
|  | I1192M | V-073 | Mosser et al. 1994/Liu et al 2012 |
|  | I1192F | WIN51711/V-073 | Mosser et al. 1994/Liu et al 2012 |
|  | V1194L | WIN51711 | Mosser et al. 1994 |
|  | M1260L | WIN51711 | Mosser et al. 1994 |
|  | A3024V | V-073 | Liu et al 2012 |
|  | S4046L | WIN51711 | Mosser et al. 1994 |
|  | P1052S | WIN51711 | Mosser et al. 1994 |
|  | N1051S | WIN51711 | Mosser et al. 1994 |
|  | A1049V | WIN51711 | Mosser et al. 1994 |
|  | A2205V | WIN51711 | Mosser et al. 1994 |
|  | V2208I | WIN51711 | Mosser et al. 1994 |
|  | I3049M | WIN51711 | Mosser et al. 1994 |
|  | T4053A | WIN51711 | Mosser et al. 1994 |
|  |  |  |  |
| HRV14 | N1100S | WIN 52035-2 | Shepard et al 1993 |
|  | N1105S | WIN 52035-2 | Shepard et al 1993 |
|  | A1150T | LPCRW_0005 | Lacroix et al. 2014 |
|  | A1150V | LPCRW_0005 | Lacroix et al. 2014 |
|  | Y1152F | Pleconaril | Ledford et al. 2005 |
|  | V1153 | WIN 52035-2 | Shepard et al 1993 |
|  | V1191L | Pleconaril | Ledford et al. 2005 |
|  | C1199Y | WIN 52084 | Heinz et al 1989 |
|  | C1199A | WIN 52084 | Heinz et al 1989 |
|  | C1199W | WIN 52084 | Heinz et al 1989 |
|  | C1199F | WIN 52084 | Heinz et al 1989 |
|  | C1119TC | WIN 52035-2. | Shepard et al 1993 |
|  | V1188M | WIN 52084/ WIN 52035-2 | Heinz et al 1989/ Shepard et al 1993 |
|  | V1188L/L1112F | WIN 52084 | Heinz et al 1989 |
|  | V1188L | WIN 52084 | Heinz et al 1989  Shepard et al 1993 |
|  | S1223G | WIN 52084, WIN 52035-2 | Heinz et al 1989 |
|  | N1105S/V1176A | WIN 52084 | Heinz et al 1989 |
|  | N1219S | WIN 52084 | Heinz et al 1989 |
|  | V1188M/C1119W |  |  |
|  |  |  |  |
| CVB3 | I1092M | Pleconaril | Groarke and Pevear 1999 |
|  | I1092L | Pleconaril | Groarke and Pevear 1999 |
|  | I1092L/L1207V | Pleconaril | Groarke and Pevear 1999 |
|  |  |  |  |
| Echovirus 11 | V117I | Pleconaril | Benschop et al 2015 |
|  | V119M | Pleconaril | Benschop et al 2015 |
|  | I183M | Pleconaril | Benschop et al 2015 |
|  | I188L | Pleconaril | Benschop et al 2015 |
